# Three-Dimensional Printing and Bioprinting in Renal Transplantation and Regenerative Medicine: Current Perspectives

**DOI:** 10.3390/jcm12206520

**Published:** 2023-10-14

**Authors:** Chrysanthos D. Christou, Stella Vasileiadou, Georgios Sotiroudis, Georgios Tsoulfas

**Affiliations:** Department of Transplantation Surgery, Hippokration General Hospital, Aristotle University of Thessaloniki, 54642 Thessaloniki, Greece; stvasileiadou@gmail.com (S.V.); george.sotiroudis@gmail.com (G.S.); tsoulfasg@gmail.com (G.T.)

**Keywords:** kidney, transplantation, 3D printing, bioprinting

## Abstract

For patients with end-stage kidney disease (ESKD), renal transplantation is the treatment of choice, constituting the most common solid organ transplantation. This study aims to provide a comprehensive review regarding the application of three-dimensional (3D) printing and bioprinting in renal transplantation and regenerative medicine. Specifically, we present studies where 3D-printed models were used in the training of surgeons through renal transplantation simulations, in patient education where patients acquire a higher understanding of their disease and the proposed operation, in the preoperative planning to facilitate decision-making, and in fabricating customized, tools and devices. Three-dimensional-printed models could transform how surgeons train by providing surgical rehearsal platforms across all surgical specialties, enabling training with tissue realism and anatomic precision. The use of 3D-printed models in renal transplantations has shown a positive impact on surgical outcomes, including the duration of the operation and the intraoperative blood loss. Regarding 3D bioprinting, the technique has shown promising results, especially in the field of microfluidic devices, with the development of tissue demonstrating proximal tubules, glomerulus, and tubuloinerstitium function, and in renal organoid development. Such models can be applied for renal disease modeling, drug development, and renal regenerative medicine.

## 1. Introduction

Renal transplantation constitutes the most commonly performed solid organ transplantation. Specifically, the Global Observatory on Donation and Transplantation estimated there were 80,926 renal transplantations (32% from living donors) conducted in 2020, accounting for 62.4% of global transplantation activity [1]. For patients with end-stage kidney disease (ESKD), renal transplantation with a living or deceased donor transplant remains the treatment of choice when compared with peritoneal dialysis or hemodialysis since it provides substantially greater quality of life and is associated with lower long-term morbidity and mortality [2]. Nevertheless, renal transplantation is still associated with various postoperative complications, including urological complications (urine leak and urinary obstruction), peritransplant fluid collections (hematomas, lymphoceles, urinomas, and abscesses), vascular complications (renal artery stenosis, renal artery thrombosis, arteriovenous fistulas and pseudoaneurysms, renal vein thrombosis), calculous disease, neoplasms, gastrointestinal complications, and herniation complications [3]. The introduction of novel technologies and the improvements in medical imaging and surgical techniques have significantly lowered the prevalence of these complications, ameliorating their negative impact on the surgical outcome.

In this new era of technological advancement, three-dimensional (3D) printing has emerged in medicine, promising to revolutionize surgical practices. Three-dimensional printing could be defined as “translating” a digital image into a 3D solid object by printing consecutive thin layers of materials [4]. Originally, 3D printing materialized in non-medical disciplines to serve the pressing demands of rapid engineering of prototypes. However, it has since expanded to other disciplines, including surgery, where 3D printing has been used for educational purposes to facilitate the comprehension of complex anatomy, for preoperative planning, and particularly for operations involving complex vasculature, for crafting customized surgical tools, and for patient counseling [5,6].

Despite the expansion of selection criteria, including “marginal” renal grafts from substandard donors, renal transplantation is limited by the shortage of transplants [7]. Specifically, in the US, only 25% of the waitlisted patients receive a transplant within five years, with patients being removed from the list due to deterioration of health or premature death [7]. Thus, the lack of donors worsens the already vast healthcare burden associated with ESKD patients on dialysis. Therefore, justifiably, kidney regeneration has been a long-standing challenge for tissue engineering. The fusion of tissue engineering and 3D printing has given rise to bioprinting [8]. This technique employs biocompatible printers and “bio-ink” to create intricate tissue structures, while the complete fabrication of functional organs remains a research objective. Bioprinting achieves the fabrication of structures of precise internal and external architecture that provide high cell viability and imitation of natural tissue features (biomimicry) [9,10].

The notion of bioengineered renal replacement therapy that will lessen the burden of dialysis is not a novel one [11,12]. Notably, bio-printable, individualized renal transplants will revolutionize renal transplantation, bringing balance to the current shortage of renal grafts. Three studies have reviewed the role of 3D printing in liver transplantation [13], the clinical value of 3D printing in renal disease [14], and kidney bioengineering strategies [15]. This study aims to provide a comprehensive review of the literature regarding the application of 3D printing and bioprinting in renal transplantation.

## 2. Search Strategy

This literature review was conducted by using the following algorithm: ((3D printing OR 3D printing OR three-dimensional printing OR rapid prototyping OR additive manufacturing) AND (kidney OR renal)). This algorithm was used in Medline and Google Scholar databases. The authors conducted the eligibility screening of the related literature independently, and a discussion among them resolved any arising disagreements.

The eligibility screening process for this study was conducted with three objectives in mind:

1. Our foremost objective was to comprehensively identify all relevant studies that employ 3D printing techniques within the context of renal transplantation.
2. A secondary aim was to encompass additional literature pertaining to skills and methodologies applicable to the field of renal transplantation.
3. Lastly, we aimed to present a comprehensive review of the existing literature on the state of bioprinting in the realm of renal regenerative medicine.

Finally, the reference lists of eligible articles were also reviewed to identify additional eligible articles. In this literature review, we included original articles written in the English language. Literature reviews, commentaries, publications of abstracts, preprints, and articles written in languages other than English were excluded. Eligibility screening was completed on the 31 July 2023.

## 3. Three-Dimensional Printing in Renal Transplantation

### 3.1. Educational Purposes

#### 3.1.1. Surgical Training—Core Skills

In Table 1, we present the studies identified by our literature review that directly tackle surgical training for renal transplantation employing 3D-printed simulation models. Uwechue et al. created a pair of recipient pelvic cavity and donor kidney graft models that utilized segments of deceased donor vessels to reproduce the actual properties and feel of real tissues [16]. A robotic surgical system was docked to the set and operated by two surgeons. Blood vessels’ anastomoses were timed, video-recorded, and tested for patency and leak resistance [16]. Claflin et al. described the development of hard-plastic right lower quadrant recipient and donor kidney graft models utilizing penrose drains as blood vessels for anastomosis training [17]. The penrose drains are cheap and can be easily replaced. Twelve surgery residents participated in a survey to assess the 3D model set following training in end-to-side anastomoses [17]. They all reported a better understanding of the anastomotic suture technique [17]. Notably, the aforementioned studies lack ureterovesical anastomosis training.

Saba et al. constructed a robotic donor nephrectomy model and a robot-assisted kidney transplantation model [18]. Three-dimensional printed molds were injected with a polyvinyl alcohol hydrogel mixture that allowed for the creation of realistic organs and tissues [18]. The kidney graft model could be perfused, cautered, and sutured. Other anatomical structures created for the nephrectomy model, except for the kidney, were the spleen, the colon, retroperitoneal fat, muscles, and the abdominal wall [18]. The recipient model included a bony pelvis, bladder, and iliac vessels. Notably, the bladder was lined with all three anatomical layers, and the 3D-printed iliac vessels were developed to match the mechanical properties of patients’ iliac vessels [18]. An experienced transplant surgeon was then evaluated in a series of four robotic nephrectomy-transplantation training simulations, including the arterial, venous, and ureterovesical anastomotic times [18]. Peri et al. developed an open renal transplantation simulation platform comprising a recipient model and a kidney graft by directly 3D printing rigid and deformable structures. Parts of the iliac vessels were replaceable and perfusable, and the bladder wall consisted of two layers [19]. Additionally, some kidney graft models included an inferior polar artery that allowed trainees to practice unifying the arteries on the bench table. Two surgical trainees completed a series of 15 simulation transplantations each, while their anastomotic time (arterial, venous, ureterovesical) was recorded, and anastomotic quality was visually rated by three experts. Although the quality of the anastomoses showed no improvement throughout the study, the arterial and venous anastomotic times were steadily shortened each time after reaching a plateau following the tenth procedure. [19]. In a separate self-assessment questionnaire, the two trainees reported higher confidence in performing the anastomoses [19]. Finally, in a recent study explicitly targeting renal transplantation, the authors developed an entirely 3D-printed, perfused robot-assisted kidney transplantation box for vascular anastomoses training [20]. A senior transplant surgeon first tested the box, which was then used to train four residents whose performance was recorded and then evaluated by senior surgeons [20]. However, the box had several limitations, most importantly, the absence of ureterovesical anastomosis simulation [20]. Notably, tangible differences were observed among the residents: their mean anastomotic time was up to 36 min for the arterial and venous anastomoses [20].

#### 3.1.2. Surgical Training—Further Skills

Renal transplantation requires high literacy in vascular handling, ligation, and suturing for anastomoses. Our literature review returned several articles that, albeit not specifically designed with transplantation in mind, could be applied in the surgical training of the aforementioned skills. Sweet et al. described the work of the Center for Research in Education and Simulation Technologies (CREST) team in devising a range of simulation and training models for endourologic applications [21]. Among them is a beating artery model developed with 3D printing techniques. This artery model has the capacity for artificial pulsation and is filled with blood that maintains the mean arterial pressure. The model is already used in laparoscopic clip application training modules [21]. Another 3D-printed model created by CREST provides laparoscopic or robotic training for vascular bleeding repair [21]. The model can simulate pulsatile or stable flow and allows blood loss calculation [21]. Vascular control is very important for managing complications in laparoscopic donor nephrectomy as well as in renal transplantation.

Postoperatively, renal-transplanted patients usually require endourological procedures. Examples include cystoscopy to remove the ureterovesical stent that is routinely placed during transplantation in many departments, percutaneous nephrostomy for obstructive hydronephrosis or pyonephrosis, and treatment for graft kidney stones—via ureteroscopy or percutaneous access. Stone removal treatment is also beneficial in pre-transplant interventions, expanding living kidney donor acceptability. Our literature review returned several articles concerning training for the aforementioned procedures, although not specifically designed for the transplanted kidney. However, we believe that the skills acquired by participants in such training simulations are readily transferable in the kidney transplantation setting. Turney et al. developed fluoroscopy-guided percutaneous nephrolithotomy-access training models by 3D printing water-soluble renal collecting systems embedded into a silicone–rubber mixture, which were then washed off and filled with contrast medium [22]. Adams et al. fabricated three distinct kidney-collecting system models by 3D printing molds injected with agarose, silicon, and polydimethylsiloxane, respectively [23]. All three models allowed clear visualization of calyces through flexible ureterorenoscopy [23]. The polydimethylsiloxane model demonstrated the greatest educational potential due to its semi-transparency, while the agarose model showed excellent ultrasound fidelity [23]. The CREST team mentioned earlier developed a 3D-printed fluoroscopy-guided percutaneous renal access model that precisely imitates needle advancement forces by using polyurethanes and silicone of varying hardness for the different layers of the human latus [21]. Tatar et al. described the work of the MedTRain3DModsim project that manufactured various training simulation stations by utilizing 3D printing and virtual and augmented reality [24]. Among these models are a standard cystoscopy/ureteroscopy and a percutaneous nephrostomy/nephrolithotomy model [24]. Urology residents assessed the usefulness and realism of the training models with a Likert scale questionnaire [24]. Trelles Guzmán et al. developed a flexible ureteroscopy training model by directly 3D printing a solid plastic block with a hollow space inside, simulating the pelvicalyceal system [25]. The model was used to familiarize residents and expert urologists with the visualization of the calyxes one by one and the removal of stones formerly placed in the model by using endoscopic baskets [25].

Apart from the urologic procedures mentioned above, there are two types of surgery for which 3D-printed training models have been designed, and the skills gained could be transferable to renal transplantation. These are laparoscopic pyeloplasty and laparoscopic or robot-assisted partial nephrectomy. The indication for pyeloplasty is pelviureteric junction obstruction, which can occur after transplantation due to stone formation or autonomic graft denervation or be present in the donor and treated concurrently with the actual transplantation [26]. Surgical training for pyeloplasty can thus be important for transplant unit staff. Poniatowski et al. described the creation of a pelviureteric junction obstruction model consisting of an enlarged pelvis and a ureter docked in a laparoscopic training box [27]. The model was cast in a 3D-printed mold using organosilicate materials. Following the completion of the pyeloplasty, trainees were evaluated on the basis of patency, leakage, and twisting at the pelviureteric junction—assessed with the aid of ultraviolet luminescent markers [27]. Lemarteleur et al. directly 3D printed kidney–pelvis–ureter models of pelviureteric obstruction using thermoplastic urethane and polyvinyl alcohol materials [28]. The seven experienced surgeons who tested these models by performing laparoscopic pyeloplasty then participated in a validity survey, rating the set for needle insertion, thread sliding, suturing strength, cutting strength, elasticity, handling, and mobility [28]. Thread sliding received the best rating and elasticity the worst. Notably, the 3D-printed model was fixed inside the laparoscopic box using springs of random stiffness to simulate tissue handling [28].

Regarding partial nephrectomy, Melnyk et al. compared the use of porcine kidneys with 3D-printed casts injected with polyvinyl alcohol in robot-assisted partial nephrectomy training [29]. The models were derived using CT scans of patients, and different polyvinyl alcohol formulations were used for testing the materials’ mechanical properties [29]. Notably, the authors achieved the development of affordable models suitable for replicating the functional properties of porcine kidneys that can be used in education, surgical planning, and procedure-specific simulations in complex urological cases [29]. Porpiglia et al. enrolled ten patients in a study aiming to evaluate the role of 3D-printed models in facilitating minimally invasive nephron-sparing surgery [30]. The cases were presented at a urology congress where attending surgeons were asked to fill out a questionnaire. The attendants scored the models for their overall usefulness, role in surgical planning, role in patient counseling, and accuracy in replicating anatomical details with scores of 8/10, 9/10, and 10/10, respectively [30].

#### 3.1.3. Patient Education

Three-dimensional printed models can be employed for patient education, contributing to a deeper comprehension of their medical condition, the intended medical intervention, and its potential complications. This, in turn, fosters improved communication and trust between healthcare professionals and patients, leading to greater collaboration and facilitating the process of obtaining informed consent. In the work of Porpiglia et al., the authors evaluated the stance of eighteen patients towards the application of 3D-printed models in their treatment [30]. The patients answered that they appreciated the use of the model during the discussion with the surgeon and that the model facilitated reaching a higher level of comprehension of their disease and the proposed intervention [30]. In a different study, Bernhard et al. enrolled prospectively seven patients with primary kidney tumors and evaluated the patient knowledge and understanding before and following the 3D model presentation as well as their satisfaction with their 3D-printed individualized model [31]. Notably, following the 3D model presentation, the patients demonstrated an improvement in understanding of kidney physiology, kidney anatomy, tumor characteristics, and the planned procedure by 16.7%, 50%, 39.3%, and 44.6%, respectively. Living donors constitute a peculiarity of transplantation where 3D printing could find a particular application. Patient-specific models could be employed to facilitate the procedure of informing the donor and the recipient by providing a tangible way to comprehend a complex procedure. 

### 3.2. Preoperative Planning

The improvement in 3D printing techniques based on anatomic imaging has led preoperative planning to a new era. Over the past years, 3D models have been used in various medical fields like urologic surgery, and now their potential use has expanded to the demanding field of transplantation. The articles that our literature review retrieved where 3D models were used solely for preoperative planning of renal transplantation are shown in Table 2. During kidney transplantation, the size and anatomical characteristics of the recipient’s pelvis play a vital role in the feasibility of surgery and, as an extension, the surgical outcome. The kidney graft must be anatomically compatible with the recipient’s pelvis, which is challenging in many cases (for example, in patients with polycystic disease where the native kidneys are significantly enlarged). Three-dimensional printed models developed for living donor kidney transplantation facilitate the prevention of potential complications during the operation by familiarizing the surgeons preoperatively with the operation at hand, thus reducing stress and increasing the surgical team’s efficiency. Kusaka et al. created a 3D kidney graft model based on the donor in two cases and a 3D pelvis cavity model based on the recipient in one case, all in anatomic size, that were used to represent the anatomical details and relationships among the anatomic structures. Surgeons performed vascular anastomoses in the special pelvic conditions of the recipient’s model and were given the opportunity to recognize and discuss the surgical anatomy with the other members of the transplantation team [32]. The authors stated that the preoperative simulation accurately mimicked the surgical procedure, facilitating the navigation of anatomical structures intraoperatively [32].

The size difference between the graft and the abdomen, the vascular anatomical variants, and the primary disease of the recipient are all critical in pediatric living donor kidney transplantation. Chadak et al., in their study, described a case in which a 3D-printed model was employed for preoperative planning in a pediatric transplantation case, leading to the avoidance of an operation with an incompatible kidney graft for the recipient in terms of size [33]. Their fabricated model represented various abdominal anatomic structures, including liver margins, the abdominal wall, native kidneys, the pelvis, and the iliac arteries and veins [33]. Through a comprehensive study of the fabricated model, the surgical team instead developed a preoperative plan and then performed a two-stage challenging operation on a two-year-old recipient [33]. In a third case, with anatomic variations of the vasculature, the authors described how the 3D-printed model aided their multidisciplinary team in identifying the various technical difficulties due to the high aortic bifurcation [33]. In all three cases, the 3D model aided preoperative planning and the decision-making procedure regarding the necessity, feasibility, ideal surgical approach, and potential complications of these procedures, which all resulted in successful transplantations without any complications [33]. Additionally, the application of 3D-printed models in case management led, in two out of three cases, to the avoidance of on-table surgical exploration of the recipient [33]. Furthermore, the authors highlight that the models facilitated the understanding of parents regarding their own and their child’s anatomy [33]. In their conclusions, the authors state that the 3D models provided a preoperative in-depth comprehension of surgical anatomy and were significantly more useful compared with 2D imaging in surgical planning [33].

Arterial calcifications are common in ESKD patients and have a crucial role in the quality of vascular anastomoses during kidney transplantation. Depending on the use of iodinated contrast, CT scans can identify specific arterial calcification features such as location, length, circumferential character, and stenosis. In pre-emptive kidney transplantation, where iodinated contrast is avoided, the preoperative assessment of arterial atheromatosis can be challenging and poses surgical risks. Three-dimensional printing could prove particularly useful in filling the gap between the preoperative assessment and physical palpation of the recipient’s arteries. Denizet et al. described a series of four cases of recipients with various degrees of arterial calcification where 3D models of the aortoiliac axis were fabricated [34]. These models presented the surgeon with the opportunity to preoperatively come in contact with the recipient’s arteries by palpation and thus assess the feasibility of the anastomosis at various potential sites of anastomosis [34]. Additionally, the model allowed the surgeon to perform a simulation of the clamping of the artery on the desired site of anastomosis [34]. The kidney transplant surgeon qualitatively assessed the models, scoring them for their visual appearance (3/10), haptic anatomy (8/10), preoperative anatomy (8/10), and whether the surgeon would use the models for preoperative planning(10/10) [34]. Therefore, the progress of 3D technologies seems to allow the reproduction of the recipient’s vessels so accurately, with high translucency and pliancy that the surgeon preoperatively could decide reliably and with high certainty the site of anastomosis, which constitutes a key part of the kidney transplantation operation.

The safety and fast recovery of living donors is a major concern in transplantation that affects the donor pool worldwide. Thus, justifiably, novel surgical techniques such as robotic-assisted nephrectomy are evolving and are being adopted by transplantation surgery as part of enhanced recovery after surgery protocols. Still, the vascular and urinary tract anatomy are the most important factors determining the suitability and the optimal surgical approach to kidney transplantation. For example, multiple renal arteries or veins could significantly affect the complexity of the transplantation process regarding both the donor nephrectomy and the renal transplantation. Thus, the collective knowledge acquired from the application of 3D printing in urology, particularly in nephrectomy, could prove equally helpful in kidney transplantation. The number of renal arteries and veins constitutes a vital factor affecting the donor nephrectomy operation and is routinely assessed preoperatively. In a recent study by Zhang et al., a total of 120 living donors and their corresponding 120 transplanted recipients were randomly divided into two groups: the 3D-printing-assisted group and the traditional operation group [35]. Notably, for the group where 3D-printed models were used during preoperative planning, the diagnostic rate of vascular variations was higher (37% vs. 18%, *p* < 0.05), the overall operation time was shorter (88.8 ± 8.2 vs. 100.4 ± 11.4 min), the amount of intraoperative bleeding was lower (79.9 ± 18.7 vs. 92.1 ± 19.4 mL, *p* < 0,05), the first-day following transplantation serum creatinine was lower (69.4 ± 14.4 μmol/L vs. 86.8 ± 12.9 μmol/L, *p* = 0.001), and their recovery time was shorter (3.7 ± 2.7 days vs. 5.1 ± 1.6 days; *p* = 0.040) [35]. Additionally, regarding donors, the serum creatinine was decreased at a higher rate in the 3D printing group compared with the traditional group (430.2 ± 134.1 μmol/L vs. 565.7 ± 193.7 μmmol/L; *p* = 0.001) [35]. The authors state that the 3D-printed models assisted the surgeon in planning a more detailed and precise operation based on patient-specific anatomic variations, performing a simulated operation on the model, avoiding unnecessary steps intraoperatively, and reaching the site of operation quickly, thus reducing the complication risk and optimizing the surgical outcome [35]. In a different study focusing on warm ischemia during partial nephrectomy, the authors highlight that the number and rate of feeding arteries in renal tumors are estimated more accurately with 3D reconstruction than with conventional CT angiography [38]. The combination of 3D visualization and 3D printing techniques is able to develop renal models of high anatomic accuracy and detail, particularly regarding the patient’s renal vasculature and urinary structures [39]. Thus, the fabrication of individualized 3D models could be used routinely during the preoperative planning in renal transplantation from living donors with significant benefit to the surgical outcome.

Three-dimensional printed kidney models are valuable tools in planning and assisting a partial nephrectomy, particularly for complex renal tumors. These physical anatomic models improve the surgeon’s understanding of anatomic details shown in imaging more tangibly and allow them to visually examine the actual anatomy preoperatively. The application of 3D-printed models in partial nephrectomy was assessed at an international urological meeting where urologists filled out questionnaires after watching live surgeries with the presentation of 3D models [40]. The participating physicians evaluated the models with positive reviews in terms of their anatomic detail and their usefulness in surgical planning [40]. Fan et al. achieved a shortened the warm ischemic time and a reduction in intraoperative blood loss during partial nephrectomies of patients with renal scores greater than eight by employing 3D-printed laparoscopic models [41].

The application of 3D printing is well established in nephron-sparing surgery in urology. Partial nephrectomy for small renal tumors provides negative margins with the preservation of renal parenchyma and, thus, significantly higher renal function with fewer postoperative complications. The decision to perform nephron-sparing surgery is based on patient and tumor factors. The location of the tumor and the relationship with the main vessels and collecting system are vital points that need to be evaluated preoperatively. Another key factor is the accurate estimation of the remaining kidney volume. In a case report study by Girón-Vallejo Ó et al. of a patient with bilateral Wilms tumor, the application of a 3D-printed model led to altering the preoperative plan, thus avoiding a radical nephrectomy since the precise estimation of the preserved renal parenchyma and the accurate reconstruction of the patient’s anatomic structure proved the feasibility of partial nephrectomy [42].

Another significant point in donor nephrectomy is the anatomy of the urinary tract, which needs careful surgical manipulations to avoid complications such as an injury in the collecting system. In a study by Kyung SY et al., postoperative urine leakage following a nephron-sparing operation was successfully predicted in 71.5% of the cases [43]. This could be attributed to the ability of 3D models to accurately provide a comprehensive understanding of the anatomy and particularly in cases of small, endophytic tumors that are in close proximity to the collecting system. In a study conducted by Wake N. et al., the role of patient-specific 3D-printed renal tumor models in the preoperative planning of complex renal mass operations was evaluated [44]. The use of 3D printing was shown to facilitate decision-making preoperatively (type of nephrectomy, surgical approach, and clamping site) [44]. Notably, the preoperative plan was altered in 30–50% of cases following the inspection of the 3D-printed model [44]. The 3D models provided enhanced confidence preoperatively cultivating a calm, non-stressful environment in the operating room, which benefits the surgical outcome [44].

Another application of 3D printing is the development of case-specific, customized surgical tools, instruments, and devices. In a recent study, a 3D-printed, patient-specific cold preservation device was developed that allowed for a laparoscopic completely intracorporeal renal autotransplantation of a patient with renal artery stenosis [36]. The cool jacket was 2 mm larger than the kidney and consisted of two sealed films that created a tunnel for the cooling agent [36]. In another study, 3D-printed models found a particular application in oncologic pelvis surgery, in providing customizing instruments that enhance the precision in the operating room and reduce the operative time [45]. Similarly customized tools could be used in renal transplantation surgeries in complex cases based on the pelvic anatomic characteristics and specifically its pelvic depth and the diameter of the iliac blood vessels. A double-J stent is typically placed during renal transplantation to protect the vesicoureteral anastomosis and facilitate urine flow. In a study by Park CJ et al., 3D-printed anti-reflux ureteral 7F double-J stents were developed with a special flap valve [46]. After a series of in vitro testing, these stents were found to prevent backward flow effectively while minimizing the reduction in forward flow [46]. Three-dimensional-printed stents with patient-specific features such as the length of the ureter or special features such as coating, holes, and anti-reflux mechanisms could be particularly useful in minimizing complications of vesicoureteral anastomosis in renal transplantations. Finally, an intriguing application of 3D printing in transplantations from a deceased donor is to maintain the integrity of the donor’s body following organ retrieval by replacing the retrieved organs with donor-specific 3D-printed organs. This application could be particularly useful in regions where organ donations following death are limited due to religious or cultural factors. In a study conducted in Taiwan, following the request of the family of an 18-year-old deceased donor, 3D-printed were employed to fulfill the family’s request [37]. Despite the time and cost associated with printing these models, such efforts should be expanded if they are proven to alter the stance toward transplantation from deceased donors.

## 4. Bioprinting in Renal Regenerative Medicine

Renal transplantation, despite being the gold standard, intriguingly it is also a halfway measure since it does not address the underlying disease while at the same time, it does not cure the patient rather than transforming and lessening the morbidity from that of chronic dialysis to the morbidity of long-term immunosuppression therapies. Except for leaving patients vulnerable to opportunistic infections and predisposing to malignancy development, immunosuppression therapy is a main alloantigen-independent factor in renal chronic allograft nephropathy [47,48]. Up to 50% of kidney transplanted patients lose the graft due to chronic allograft nephropathy within ten years from transplantation [49]. Kidneys are particularly complex organs with more than 20 different cell types [50]. Bioprinting a kidney whose cell lines retain their viability and functionality long-term is a herculean task. The 3D bioprinting approach holds potential due to its ability to achieve detailed structures, which may lead to better biomimicry [51]. For organ 3D bioprinting, two different strategies have emerged: scaffold-based and scaffold-free [52]. While revolutionary, 3D bioprinting is still in its foundational stages, especially concerning the production of complex structures. Some of the primary challenges include ensuring vascularization, creating a functional nephron unit, and addressing the intricate balance of cellular interactions. Additionally, the issue of scalability and reproducibility across different bioprinting platforms poses significant hurdles. Currently, the field is seeing advancements primarily in microfluidic device development that demonstrate renal function, which represents a more immediate and tangible step towards replicating kidney function. In this section, we have identified and present the related literature where 3D bioprinting is employed in developing renal structures.

Table 3 summarizes the identified studies where 3D bioprinting was employed in the development of renal cultures/tissues. The first study utilizing 3D bioprinting to develop convoluted renal proximal tubules in vitro was published in 2016 [53]. Homan KA Et al. developed perfusable microfluidic-based chips that housed renal proximal tubules that were fully embedded in an extracellular matrix [53]. The proximal tubules were characterized by an open-lumen architecture, which was circumscribed by proximal tubule epithelial cells that maintained cell viability and functionality for over two months [53]. During printing, a fugitive ink (containing a triblock copolymer of polyethylene-polypropylene-polyethylene and thrombin) was used that was then removed before cell seeding. Gene expression analysis of 33 key proximal tubule epithelial cells genes revealed cells that these cells were transcriptionally similar to primary renal proximal tubule epithelial cells [53]. Finally, the researchers demonstrated how their model could be used to investigate drug-induced tubule damage mechanisms by successfully inducing dose-dependent tubular damage using cyclosporine A [53]. Notably, their model lacked vasculature, limiting its application in renal reabsorption studies. In 2019, researchers from the same department published a study aiming to develop a 3D bioprinted a microfluidic-based vascularized proximal tubules model, embedded in extracellular matrix, to investigate the reabsorption of solutes via tubular-vascular exchange [54]. Notably, the markers observed confirmed the presence of endothelial tissue and the perfused model demonstrated active reabsorption of albumin and glucose [54]. Additionally, the authors explored the role of the model in disease modeling by inducing hyperglycemic conditions and monitoring endothelial cell dysfunction [54].

Proximal tubules constitute the main site of nephrotoxicity, and thus, 3D bioprinted renal proximal tubule constructs could be applied during drug screening. In a study by King SM et al., the role of 3D bioprinting in producing constructs regarding drug-induced tubular damage was further investigated by developing fully cellular human in vitro proximal tubule interstitial interface that consisted of primary human renal proximal tubule epithelial cells supported by interstitial cell types including fibroblast and endothelial cells aiming to provide a microenvironment that supports the health and function of the polarized epithelia [55]. Notably, following 30 days of culture, the tissues demonstrated sufficient metabolic activity with stable levels of expression of many important renal transporters and a viable intrarenal renin-angiotensin system [55]. In addition, the 3D-printed tubules demonstrated cisplatin-depended nephrotoxicity and a TGFβ-induced fibrotic response [55]. Such models could be employed in the early stages of the drug development pipeline to reduce the occurrence of costly failures at the late stages of development. In addition, it is highlighted that the choice of the bio-ink that will encapsulate the 3D bioprinted cells is crucial in their long-term viability and functionality [61].

In a study, Addario G. et al. aimed to develop microfluidic-based renal tubulointerstitium models for in-vitro studies employing primary murine tubular cells, endothelial cells, and fibroblasts using a microfluidic 3D bioprinter [57]. The effect of different materials of the bio-ink was investigated, with a recorded cell viability on day 7 of >91% and >82%, for alginate-based and pectin-based bio-ink, respectively [57]. Limited growth and gradual death of endothelial cells was observed when cultured in a medium lacking the vascular endothelial growth factor, highlighting the essential role of the bio-ink in the support and maturation of the cell lines used. In a different study by Ali M et al., the role of porcine kidney extracellular matrix-derived bio-ink in facilitating renal tissue formation and maturation was investigated [56]. Initially, the porcine kidneys were decellularized while the extracellular matrix was preserved. Then, the matrices went through solubilization and methcrylation to derive photo-crosslinkable hydrogels [56]. The derived hydrogels were tested using a Quantibody Growth Factor Array, which revealed that despite the processing, the hydrogels maintained a plethora of cytokines and growth factors [56]. The hydrogels were used to formulate a bio-ink, which was then tested for its ability to support the cell viability, proliferation, and adhesion of human primary kidney cells [56]. The bio-ink allowed for a high proliferation with an increase in the number of cells on days 1, 3, and 5 of cell cultures, whereas in the control group (gelatin methacrylate was used), a gradual decrease in the number of cells was observed. Additionally, the cell viability was higher than 95% [56]. Finally, 3D-bioprinted renal constructs were developed, mixing the human primary kidney cells with the derived bio-ink. Notably, a 90% cell viability at day 14 was observed, while at the same time the bioprinted construct demonstrated at day 14 a significant amount of sodium uptake and significantly higher hydrolase activity when compared to control constructs [56].

3D bioprinted renal organoids can be fabricated for renal disease models, during drug development and screening, and in renal regenerating medicine. In a study by Lawlor TK et al., the role of cellular extrusion bioprinting was explored in providing rapid and high throughput generation of kidney organoids with high cell viability [58]. Employing human pluripotent stem cells, they manage to produce 3D-printed organoids that, within 20 days of culture, formed nephrons with the presence of podocytes, proximal tubules, distal tubules, loop of Henle thick ascending limb, connecting segments, and additional cellular components including endothelial cells and renal stroma [58]. The authors investigated how changing various bioprinting parameters, including well format, the speed of tip movement for a given rate of cell extrusion, and the organoid conformation, affect the properties of the resulting organoids in terms of tissue thickness, coefficient of differentiation, and nephron number [58]. Notably, changing to a 3D bioprinted line conformation demonstrated elevated nephron number [58]. The authors evaluated cell viability following aminoglycoside use, which significantly decreased providing drug-induced nephrotoxicity [58]. Renal organoids have been proven more effective in predicting drug-induced nephrotoxicity compared to 2D cultures of renal proximal tubule epithelial cells due to their rapid differentiation and loss of key transporters and metabolic enzymes [62,63]. Finally, the authors managed to generate a kidney patch that contained 4 × 10^5^ cells across a total field of approximately 4.8 × 6 mm [58]. Studies have reported the vascularization and maturation of such organoids following transplantation under the renal capsules of mice [64].

Therefore, except for bioprinting transplantable kidneys, 3D bioprinting could be used in the management of ESKD in regenerative medicine by partially restoring renal function. Intriguingly, restoring as little as ten percent of the renal function could allow patients with ESKD to disengage from dialysis, significantly improving their quality of life [65]. In a recent study, Jo H. et al. developed an autologous omentum patch to investigate its role in the treatment of ESKD [59]. Specifically, the authors investigated the effect of transplanting the omentum patch in the renal subcapsular space of rats suffering from unilateral ureter obstruction-induced kidney injury [59]. Initially, the authors utilized autologous omentum tissue, fibrinogen, and thrombin to fabricate two bio-inks [59]. An artificial intelligence tool generated the omentum patches design, printed using a bioprinter [59]. Two weeks after transplantation, renal tubular damage, and fibrosis-related gene expression were measured [59]. In the omentum patch group, decreased tubular damage and under-regulation of fibrotic mechanisms were observed compared to a group of rats transplanted with the fibrin patch group [59]. In a different study by Singh KN et al., the therapeutic role of transplanting vascularized tubular renal tissue in a chronic renal disease model was investigated [60]. Initially, porcine kidneys were decellularized and lyophilized to prepare a solution of extracellular matrices, then mixed with sodium alginate to produce a hybrid bio-ink [60]. Along with the bio-ink, human bone marrow-derived mesenchymal stem cells, renal proximal tubular epithelial cells, and human umbilical vein endothelial cells were used in 3D coaxial bioprinting of monolayer and bilayer complex hollow structures [60]. Following four weeks of culture using vascularized renal proximal tubule-on-a-chip conditions, the grafts were transplanted into the renal subcapsular part of unilateral ureter obstruction-modeled immunodeficient mice [60]. Two weeks following transplantation, the unilateral ureter obstruction transplanted models demonstrated decreased expression of alpha smooth-muscle actin and elevated expression of aquaporin 1 compared with the non-transplanted models and also expression of markers indicating neovascularization [60].

## 5. Discussion

In this review, we first comprehensively reviewed studies where 3D-printed models were employed in renal transplantation. Specifically, we identified studies where 3D-printed models were used in the training of surgeons through renal transplantation simulations [16,17,18,19,20], in the preoperative planning of donor nephrectomy [35], pediatric renal transplantations [33], autotransplantation [36], and adult renal transplantations [32,34,35], and in organ retrievals [37]. Depending on the complexity of the model and the printing methodology, the printing time and the cost of these models greatly varied from four hours to 15 h and from $95 up to €5000, respectively. Notably, only one out of the five studies regarding the application of 3D-printed models for training recorded the printing time, while five out of six studies recorded the printing time when 3D-printed models were used in preoperative planning. This highlights the significance of printing time in time-sensitive situations. Long printing times currently severely limit the application of 3D printing in emergencies.

Additionally, we presented a series of studies where 3D-printed models are employed for surgical training that we believe can be extrapolated into renal transplantation. Specifically, various models for surgical training, including a series of 3D-models models by CREST for endourological procedures training [21], fluoroscopy-guided percutaneous nephrolithotomy-access training models [22], models for flexible ureterorenoscopy [23,25], the MedTRain3DModsim project, which includes models for cystoscopy/ureteroscopy and for percutaneous nephrostomy/nephrolithotomy [24], laparoscopic pyeloplasty [26,27,28], and robot-assisted partial nephrectomy [29,30]. We believe that the skills acquired by the above models are transferable and can be of value in renal transplantations. 

Furthermore, we presented a series of studies that focus on the application of 3D-printed models in the management of renal diseases that we believe could find applications in renal transplantation. Specifically, 3D models have effectively been used in minimally-invasive nephron-sparing procedures to expand the patients’ comprehension of their disease, the proposed operation, and the potential complication, thus facilitating the informed consent procedure [30,31]. Finally, we highlighted how 3D-printed could be used to fabricate customized, patient-specific tools and instruments. Current studies include the fabrication of a 3D-printed cold preservation device that facilitated a laparoscopic intracorporeal renal autotransplantation [36], the development of 3D-printed customized surgical tools in oncologic pelvis surgery cases [45], and the fabrication of a 3D-printed double-J stent with a flap valve [46]. These studies demonstrate how 3D printing could lead to customized, individualized care in renal transplantation based on the characteristics of the patient, such as the pelvic depth, the diameter of the iliac blood vessels, and the length of the kidney’s ureter.

As a specialized surgical field, transplantation training is not formalized in many countries [66]. The training of residents is primarily based on steadily increasing responsibilities through which residents gain surgical skills and experience. Thus, the training of residents is based on a trade-off between the need of residents for hands-on experience and the need for high-quality care and optimal surgical outcome. Studies have described adequate results with less experienced surgeons perform under senior transplant surgeons supervision [66,67].Nevertheless, trainee training in transplantation has clearly space for growth. Except for 3D printing, the role of virtual reality in facilitating training in transplantation has been investigated [68,69]. Similar to 3D printing, virtual reality can be used to perform patient-specific preoperative surgery rehearsals to aid physicians in familiarizing themselves with the patient-specific anatomy, designing a surgical plan, and practicing before the actual operation [70]. Nevertheless, virtual reality lacks compared to 3D printing since it lacks tactile feedback and physical interaction, which are crucial for surgical skill development.

Thus, 3D-printed models could transform how medical students and junior doctors learn and train, entirely replacing cadavers by providing surgical rehearsal platforms across all surgical specialties that enable training with tissue realism and anatomic precision [71]. In renal transplantation, 3D-printed simulation models could replace the first cases of the learning curve of a new resident or a surgeon unfamiliar with renal transplantations, becoming more familiarized with the surgical field before engaging in actual operations. In this simulated environment, the surgeon can be evaluated in their ability to perform the vascular anastomoses and receive feedback in a safe, non-stressful environment. Additionally, 3D-printed renal models could be of value even for experienced transplant surgeons in complex cases with vascular variations for preoperative planning. As highlighted in the studies presented, the use of 3D-printed models in renal transplantations has shown a positive impact on the surgical outcome, including decision-making regarding a feasible surgical plan, the duration of the operation, the intraoperative blood loss, and the serum creatinine level at the first day following transplantation.

ESKD is approaching pandemic proportions, which is worsening the disequilibrium between available grafts and the demand for transplantable organs [72]. The application of regenerative medicine and bioengineering, including 3D bioprinting, could lead to a new era in renal transplantation. As shown in our review, 3D bioprinting has already shown promising results, especially in the field of microfluidic devices, with the development of tissues demonstrating proximal tubules [53,54,55,56,60], glomerulus [56], and tubuloinerstitium [57] functions. Such models could be applied in renal disease modeling and during drug development for nephrotoxicity investigations. Finally, focusing on transplantation, studies employing 3D bioprintable tissues for the management of ESKD have demonstrated promising results in animal models, restoring part of the renal function [59,60].

Alternative promising approaches for the management of ESKD are the use of wearable and implantable artificial kidney devices and xenotransplantation. Wearable hemodialysis devices have achieved proof-of-concept in human clinical trials [73,74], while implantable hemodialysis devices have not yet reached human trials [75]. Wearable hemodialysis devices aim to provide continuous renal replacement therapy, achieving higher solute clearance than standard hemodialysis. While wearable and implantable artificial kidney devices demonstrate promising results and, in terms of scalability, could be the most practical approach for ESKD management, they still face several challenges, including the engineering challenge of miniaturizing the devices, optimizing sorbent materials, patient suitability and accessibility, preventive anticoagulation for long-term patency, microbiological contamination, and long-term effectiveness [76].

Renal xenotransplantation of genetically engineered pigs for human xenotransplantation has, on the other hand, already reached pre-clinical phases and is closer to addressing the graft shortage compared with 3D bioprinting, where the research is still at a foundational stage. Specifically, in a recent study, Porrett et al. performed bilateral native nephrectomies in a human brain-dead decedent and then transplanted two bioengineered renal grafts [77]. Notably, the decedent remained hemodynamically stable through reperfusion; no hyperacute rejection or porcine virus transmission was observed, while the kidneys retained viability until termination 74 h later [77]. In a different study by Montgomery et al., genetically engineered pig kidneys were transplanted into two brain-dead human recipients, demonstrating urine and creatinine output following reperfusion without signs of hyperacute rejection [78]. Nevertheless, many challenges are still associated with renal xenotransplantation, including long-term viability and functionality, immunological barriers, the risk of zoonotic diseases, ethical and moral concerns, public acceptance, cost, and accessibility. Despite the highlighted benefits of the application of 3D printing in renal transplantation, we acknowledge that it is currently applied at the research level rather than routinely in the clinical setting. Cost remains the main constraint in the widespread application of 3D printing in the clinical setting. Most of the studies in our review employed extrusion-based microsystems to develop their models, which are generally the most affordable among the 3D printing methodologies. However, notably, these studies originated from high-income countries, raising concerns about the accessibility of middle- and low-income countries to these technologies. Nevertheless, we believe that as the cost associated with 3D printing steadily decreases, the use and application of 3D printing in healthcare, particularly in renal transplantation, will significantly expand in the upcoming years to middle- and low-income countries [79]. The danger remains, however, that 3D printing and bioprinting will be just one more brick in the wall of inequality. Finally, an intriguing point for the future is how all these emerging state-of-the-art technologies, including 3D printing and bioprinting, artificial intelligence, robotics, virtual and augmented reality, and genomics, will interact, shaping the clinical setting of the future.

## Figures and Tables

**Table 1:** Studies employing 3D printing in the surgical training of renal transplantation.

	First Author	Imaging Modality	Printer Type/Employed Materials	Number of 3D-Printed Models	Printing Time	Cost (per Model)	Aim
1.	Uwechue R. [16]	Abdominal and pelvic CT imaging	NR	Two (donor and recipient)	NR	GBP 1000	Two robotic surgeons performed vascular anastomoses between the donor’s and recipient’s iliac vessels.
2.	Claflin J. [17]	Abdominal and pelvic CT imaging	PrintrBot (©PrintrBot, Lincoln, California)/Polylactic acid filament	One	NR	USD 178	Residents were trained to perform end-to-side arterial and vein anastomoses with the instruction of an attending transplant surgeon.
3.	Saba P. [18]	Abdominal and pelvic CT imaging	Fusion3 F400-S (©Fusion3 Design, Greensboro, NC)/PVA hydrogel mixture	Two (donor and recipient)	NR	USD 95 worth of materials	A certified transplant surgeon completed a robotic training curriculum, including four RAKT simulation procedures
4.	Peri A. [19]	Abdominal and pelvic CT and MRI imaging	Objet260 Connex 3 (©Stratasys, Eden Prairie, MN, USA)/Combination of rigid and deformable materials (photo-rather polymeric resins)	35 (five pilot models)	15 h for each procedure	EUR 2665 for the fixed platform plus EUR 220 for each procedure	Two surgical trainees completed a series of 15 simulation transplantations each while their anastomotic time was recorded, and three experts visually rated anastomotic quality.
5.	Campi R. [20]	Abdominal and pelvic CT imaging	FDM printer/Combination of poly(lactic acid), silicon elastomer, and soft materials	One box	NR	EUR 5000 for the box and EUR 100 for disposable vessels	Four surgical trainees performed training sessions with the RAKT box, performing vascular anastomoses between the graft’s renal and recipient’s iliac vessels.

Abbreviations: CT: computed tomography, FDM: fused deposition modelling, MRI: magnetic resonance imaging, NR: not recorded, PVA: polyvinyl alcohol, RAKT: robot-assisted kidney transplantation.

**Table 2:** Studies employing 3D printing in the preoperative planning of renal transplantation.

	First Author	Imaging Modality	Printer Type/Employed Materials	Number of 3D-Printed Models	Printing Time	Cost (per Model)	Aim
1.	Kusaka M. [32]	Abdominal and pelvic CT imaging	Objet500 Connex 3 (©Stratasys, Eden Prairie, MN, USA)/VeroClear, VeroMagenta, VeroCyan, TangoPlus	Two graft models and one recipient model	NR	NR	Surgeons performed preoperatively vascular anastomoses in patient-specific models and discussed the results with the transplant team.
2.	Chandak P. [33]	Abdominal and pelvic CT and MRI imaging	Objet500 Connex1 (©Objet-Stratasys, Rehovot, Israel)/Acrylic photopolymers	Three	9 h	USD 1500 per case	Preoperative planning and decision-making (regarding feasibility) in three complex pediatric renal transplantation cases
3.	Denizet G. [34]	Abdominal and pelvic CT without contrast	Multi-jet printer (©Scalia, Cryla Group, Besancon, France)/Elastomeric resin and acrylonitrile butadiene styrene	Four	10–14 h	EUR 300–400	Assess the feasibility of the anastomosis at various potential sites of anastomosis by palpation—simulation of clamping.
4.	Zhang J. [35]	CT angiography and CT urography	NR/Photoreceptor resin	60	4–6 h	NR	Assist preoperative planning, including assessing vascular variations, performing simulations of the planned operation, and deciding on the surgical site of living donor renal transplantations.
5.	Cui D. [36]	Abdominal and pelvic CT imaging	©Shanghai liantai Technology Co., Ltd. rs4500 China/PLA filament	One	8 h	NR	Development of a 3D-printed cold preservation device that makes feasible the laparoscopic intracorporeal renal autotransplantation
6.	Weng JY. [37]	Use of a standard template	FreeDMake (©Blooming Electronics Co Ltd., Taiwan)Polylactic acid	Four	6 h 35 min	USD 4 per hour printing	Maintain body integrity following organ retrieval as psychological support to the family. Alter the stance toward transplantation from a deceased donor.

Abbreviations: CT: computed tomography, MRI: magnetic resonance imaging, NR: not recorded, PLA: polylactide.

**Table 3:** Studies developing 3D-bioprinted renal models.

	First Author	Cell Lines-Subjects	Printer Type/Bioink	Printing Strategy	Aim	Results
1.	Homan KA. [53]	PTEC	AGB 10000, (©Aerotech Inc., Pittsburgh, PA, USA)/gelatin-fibrin hydrogel, fugitive ink, silicone elastomer	Scaffold based	Develop 3D convoluted renal proximal tubules within microfluidic chips	The microfluidic-based model showed high cell viability, gene expression pattern close to primary renal PTEC, and superior functional albumin uptake compared with 2D controls
2.	Lin NYC. [54]	PTEC, vascular endothelial cells	3D-Bioplotter (©EnvisionTEC)/gelatin-fibrin-based ECM, fugitive ink	Scaffold based	Vascularized proximal tubules (microfluidic platform) demonstrating reabsorption of solutes (tubular-vascular exchange)	The model demonstrated active albumin and glucose reabsorption.
3.	King MS [55]	HUVEC, adult, renal, fibroblast, and renal PTEC	NovoGen Bioprinter Instrument (©Organovo Inc., San Diego, CA, USA)/NovoGel Bio-Ink	Scaffold based	Develop a renal proximal tubule model in vitro supported by renal fibroblast and endothelial cells.	The model demonstrated functions of the native proximal tubule, drug-induced nephrotoxicity, and renal fibrosis.
4.	Ali M. [56]	Porcine kidneys/human primary kidney cells	ITOP system/KdECMMA-based	Scaffold based	Investigate the role of KdECMMA-based bio-ink in supporting 3D bioprinted renal constructs from human primary kidney cells	The constructs demonstrated high cell viability, and significantly higher sodium reabsorption and hydrolase activity compared to the control group.
5.	Addario G. [57]	pmTEC, HUVEC fibroblasts	Microfluidic bioprinter (©RX1 Aspect Biosystems, Canada)/alginate, gelatin, pectin	Scaffold based	Development of a microfluidic-based tubulointerstitium model for in-vitro studies	The authors achieved to develop multiple models of different cell-line/bio-ink formulations comparing the cell viability and metabolic activity of the various constructs
6.	Lawlor KT. [58]	hPSCs	NovoGen MMX extrusion-based 3D cellular bioprinter (©Organovo Inc., San Diego, CA, USA)/Cellular Bio-Ink.	Scaffold free	Develop renal organoids of highly reproducible cell number and viability by extrusion-based 3D cellular bioprinting.	Achieved the formation of renal organoids demonstrating a high resemblance to nephron histology, high reproducibility/cell viability, and drug-induced nephrotoxicity
7.	Jo H. [59]	Autologous omentum tissue/UUO Rats	Dr. INVIVO (©ROKIT Healthcare, Inc., Seoul, Korea)/fibrinogen, thrombin	Scaffold free	Transplantation of an autologous omentum patch in the renal subcapsular space for immune regulation and tissue regeneration	Reduced tubular injury and downregulation of fibrosis-inducing mechanisms were observed in the omentum patch group.
8.	Singh NK. [60]	Porcine kidneys, hBMMSC, renal PTEC, and HUVEC, UUO mice	In-house developed 3D cell-printing system/decellularized ECMs, alginate, pluronic	Scaffold based	Develop a 3D microfluidic vascularized renal tubular tissue-on-a-chip. Transplant grafts in UUO mice	Perfusable tubular constructs were developed with the ability to switch between monolayer and bilayer. Markers of tissue maturation were observed regarding renal tubular tissue and vascular tissue. UUO

Abbreviations. ECMs: extracellular matrices, hBMMSC: human bone marrow-derived mesenchymal stem cells, hPSCs: human pluripotent stem cells, HUVEC: human umbilical vein endothelial cells, ITOP: integrated tissue-organ printing, KdECMMA: photo-crosslinkable kidney extracellular matrix, pmTEC: primary murine tubular epithelial cells, PTEC: proximal tubule epithelial cells, UUO: unilateral ureter obstruction.

## Data Availability

No new data were created or analyzed in this study. Data sharing is not applicable to this article.
